# The pregnancy-related anxiety characteristics in women with gestational diabetes mellitus: why should we care?

**DOI:** 10.1186/s12884-021-03887-2

**Published:** 2021-06-10

**Authors:** Feng Fu, Ping Yan, Shuping You, Xinmin Mao, Tingting Qiao, Li Fu, Yanni Wang, Yali Dai, Palida Maimaiti

**Affiliations:** 1 School of Nursing, Xinjiang Medical University, No. 567, Shangde North Road, Urumqi, 830000 Xinjiang China; 2 Reproductive Medicine Center, The First Affiliated Hospital of Xinjiang Medical University, Urumqi, China; 3 Emergency Trauma Center, The First Affiliated Hospital of Xinjiang Medical University, Urumqi, China

**Keywords:** Anxiety, Gestational diabetes mellitus, Pregnancy, Care, Management

## Abstract

**Background:**

Gestational diabetes mellitus (GDM) is very commonly-seen in clinical settings, and GDM patients may have higher levels of anxiety. It’s necessary to evaluate the anxiety level and potentially influencing factors in patients with GDM, to provide insights for the management of anxiety of GDM patients.

**Methods:**

Patients with GDM treated in our hospital from May, 2018 to May, 2020 were included. We evaluated the characteristics of patients and the scores of pregnancy-related anxiety scale for anxiety level, vulnerable personality style questionnaire (VPSQ) for personality, general self-efficacy scale (GSES) for self-efficacy, social support rating scale (SSRS) for social support level. Logistic regression analyses were conducted to identify the potential influencing factors of anxiety in GDM patients.

**Results:**

A total of 386 GDM patients were included, the incidence of anxiety in patients with GDM was 59.07%. Anxiety was positively correlated with the susceptible personality (*r* = 0.604, *p* = 0.023), and it was negatively correlated with self-efficacy and social support (*r* = -0.586 and -0.598 respectively, all *p* < 0.05). The education level, monthly income, abnormal pregnancy (miscarriage, premature rupture of membranes) and cesarean section history and first pregnancy were the independent influencing factors for the anxiety in the patients with GDM (all *p* < 0.05).

**Conclusions:**

The anxiety of GDM patients is very common, early care and interventions are warranted for those patients with abnormal pregnancy and cesarean section history, first pregnancy, lower education level, and less monthly income.

## Background

Gestational diabetes mellitus (GDM) refers to the first occurrence or discovery of impaired glucose tolerance and diabetes during pregnancy [1]. According to reports [2, 3], the global prevalence of GDM varies from 2.4% to 22.3% among pregnant women, and the prevalence of GDM in China is 9.3% to 17.1%. It has been reported that compared with normal pregnant women, the incidence of pregnancy-induced hypertension, hyperamniotic fluid, neonatal hypoglycemia, and respiratory distress in GDM patients is significantly higher, and the incidence of cesarean section in GDM pregnant women is 1.58 times that of normal pregnant women [4]. Meanwhile, the intrauterine hyperglycemia environment of GDM patients can increase the risk of obesity and diabetes in the offspring [5]. Therefore, effective management of GDM patients is an urgent problem to be solved.

The results of previous studies [6, 7] have shown that more than 40% of GDM patients are accompanied by anxiety. Besides, studies [8, 9] have shown that negative emotions such as anxiety and depression cause the sympathetic nerves to release glucagon, which in turn increases the patient's blood sugar level and affects the patient's blood sugar control. Therefore, if the anxiety level of GDM patients is not improved, it is not conducive to blood sugar control, which may affect the pregnancy outcome. Currently, the pregnancy-related anxiety characteristics and influencing factors in patients with GDM remain unclear, it’s necessary to understand the anxiety level and potentially influencing factors in patients with GDM. In this present study, we aimed to evaluate the pregnancy-related anxiety characteristics and influencing factors in patients with GDM, to provide insights into the management of GDM. We hypothesized that the incidence of anxiety in patients with GDM was high, and anxiety level was associated with many factors such as the education level, income, abnormal pregnancy et al.

## Methods

### Human subjects

This study protocol had been reviewed and approved by the ethical committee of Xinjiang Medical University hospital (approval number: 20180349), and written informed consents had been obtained from all the participants.

Pregnant patients with GDM treated in our hospital from May, 2018 to May, 2020 were considered for inclusion. The patients were included if they met all of following inclusion criteria: (1) routine oral glucose tolerance test screening was performed between 24 and 28 weeks of gestation, and the GDM diagnosis met the related diagnostic criteria [10, 11]; (2) adult patients with age ≥ 20 years; (3) patients were well-informed and agreed to participant in our study. The exclusion criteria were: (1) pregnant patients with other complications including hypertension, pre-eclampsia, pneumonia, fever; (2) patients who was cognitively impaired or deaf or mute, which might have biases on the survey results; (3) patients who did not agree to participant in our study.

### Data collection

Following Chinese research tools were used for data collection.

Questionnaire on the general condition -This questionnaire was used to investigate the demographic and sociological information of the patient, including the patient’s age, education level, occupation, monthly family income, whether there is a history of abnormal pregnancy or cesarean section.

Pregnancy-related anxiety scale -This scale was compiled by scholar Xiao et al. [12–14], and it included 13 items and three subscales, namely concerns about patient herself, worry about fetal health, and worry about childbirth. It used a Likert 4-level scoring method of 1 to 4 points, 4 indicating strongly agree, 3 indicating agree, 2 indicating disagree, 1 indicating strongly disagree. Cronbach's α coefficient and the test–retest reliability was 0.81 and 0.79 respectively. A score ≥ 24 indicated pregnancy-related anxiety.

Vulnerable Personality Style Questionnaire (VPSQ)—The questionnaire contained 9 items, two subscales with a Likert 5-level method of 1 to 5 points [15]. Patient was separated from susceptibility (> 15 points) and non-susceptibility personality (≤ 15 points). The Cronbach's α coefficients of the total scale and susceptibility subscale were 0.651 and 0.776 respectively.

General Self Efficacy Scale (GSES)—This scale was compiled by Zhang et al. [16] with a total of ten items, and each item was scored as 1 to 4 points from "completely incorrect" to "completely correct". The scale is a one-dimensional scale with a total score of the sum of items. The Cronbach's α coefficients of the total scale was 0.669. A higher score indicated a higher level of self-efficacy.

Social Support-Rating Scale (SSRS)—This scale had ten items with three dimensions, including the utilization of objective support, subjective support, and social support [17]. The Cronbach's α coefficients of the total scale and susceptibility subscale were 0.683 and 0.614 respectively. The higher the value, the higher the social support patients had.

### Survey process

The questionnaire survey process was approved by our hospital and the included patients. The surveys were distributed between the dates of May 2018 to May 2020, and generally it took 15 ~ 25 min to complete the survey. All questionnaires were distributed face-to-face by two of our authors with uniform instructions. For those patients who could not complete the questionnaire alone, the investigators would assist them for the interpretation of questions, and no instructive or biased suggestions were provided during the decision process of patients. Any question about the questionnaire was answered on the spot. We checked for omissions in time to ensure the completeness and accuracy of collected information. All the questionnaires had been given unique identity number for data management.

### Statistical analysis

All the data were analyzed with SPSS 23.0 software. We conducted descriptive analyses on the characteristics and scores of respondents. The statistical descriptions were presented as frequency or rate, χ^2^ tests were used for group comparison. The continuous values were presented as mean and standard deviation, and t tests or ANOVA tests were performed for data comparison. Spearman rank correlation and Pearson correlation analysis were performed to evaluate the characteristics of elders and related scores based on the data characteristics. Logistic regression analyses were conducted to identify the potential risk factors for anxiety in patients with GDM. In this present study, the difference was considered as being statistically significant if *p* < 0.05, and all of the tests were two-sided.

## Results

### The characteristics of included patients

We firstly identified 400 potential patients for this survey and received 386 qualified answers, with an overall response rate of 96.50%. The general characteristics of patients were presented in Table 1, the average anxiety score for included participant was 24.20 ± 6.81, the anxiety score of 228 patients were higher than 24.00, thereby the incidence of anxiety in patients with GDM was 59.07%, indicating that most included patients had higher level of anxiety.

**Table 1:** The characteristics of included patients

Items	Mean ± standard deviation
Age(y)	28.94 ± 5.28
Gestational age(w)	32.15 ± 4.42
Average monthly income (RMB)	6841.99 ± 375.16
Anxiety score	24.20 ± 6.81
VPSQ score	15.98 ± 4.25
GSES score	30.19 ± 6.18
SSRS score	22.43 ± 4.63

Notes: *VPSQ* vulnerable personality style questionnaire, *GSES *general self-efficacy scale, *SSRS *social support rating scale

We further conducted the univariate analyses on the pregnancy-related anxiety in patients with gestational diabetes. As shown in Table 2, there were significant differences on the anxiety level in the patients with different education level, occupation, monthly income, abnormal pregnancy history, cesarean section history and first pregnancy (all *p* < 0.05), yet there was no significant difference on the anxiety level in patients with different age (*p* = 0.064).

**Table 2:** Univariate analysis on the pregnancy-related anxiety in patients with gestational diabetes patients

Items	Cases	Anxiety level	t/F	*p*
Age				
< 30y	246	24.27 ± 6.31	5.374	0.064
30-40y	109	24.02 ± 5.98		
> 40y	31	24.19 ± 6.14		
Education level				
Primary school	46	29.07 ± 4.18	4.136	0.008
Junior school	88	26.13 ± 4.26		
Senior school	108	23.94 ± 5.03		
College	144	22.07 ± 5.85		
Occupation				
Business and services	138	24.43 ± 6.28	6.102	0.024
Farming	165	24.17 ± 5.04		
Others	83	24.13 ± 6.72		
Monthly income (RMB)				
< 3000	31	26.22 ± 4.21	3.875	0.045
3000 ~ 6000	148	24.64 ± 5.96		
60,001 ~ 9000	175	24.18 ± 5.25		
> 9000	32	24.13 ± 6.08		
Abnormal pregnancy history				
Yes	28	26.24 ± 6.10	3.025	0.017
No	358	24.12 ± 5.38		
Cesarean section history				
Yes	67	26.39 ± 7.16	2.162	0.009
No	319	24.13 ± 5.44		
First pregnancy				
Yes	231	24.68 ± 6.13	5.107	0.012
No	155	24.02 ± 5.27		

Notes: *t* t test, *F* ANOVA test

### The analysis of the correlation between anxiety and VPSQ, GSES, SSRS

All the data were normally distributed, the analysis of the correlation between pregnancy-related anxiety and susceptible personality, self-efficacy, and social support in GDM patients showed that pregnancy-related anxiety was positively correlated with the susceptible personality (*r* = 0.604, *p* = 0.023), and it was negatively correlated with self-efficacy and social support (*r* = -0.586 and -0.598 respectively, all *p* < 0.05).

### Influencing factors for anxiety in the patients with GDM

We used the anxiety as the dependent variable, and the positive results of Table 2 as the independent variable to conduct the logistic regression analysis. As presented in Table 3, the education level, monthly income, abnormal pregnancy and cesarean section history and first pregnancy were the independent influencing factors for the anxiety in the patients with GDM (all *p* < 0.05), indicating that the GDM patients with abnormal pregnancy and cesarean section history, first pregnancy, lower education level, and less monthly income had higher risk for anxiety.

**Table 3:** Logistic regression analysis on the influencing factors for anxiety in the patients with GDM

Variables	β	SE	OR	95%CI	*p*
Abnormal pregnancy history					
Yes	0.143	0.106	2.964	1.130 ~ 4.355	0.002
Cesarean section history					
Yes	0.120	0.129	1.742	1.112 ~ 2.941	0.023
First pregnancy					
Yes	0.033	0.121	1.608	1.324 ~ 2.973	0.036
Education level					
Primary school	0.458	0.650	1.601	1.114 ~ 1.903	0.027
Junior school	0.302	0.273	1.213	1.042 ~ 1.988	0.029
Senior school	0.918	0.188	0.593	0.103 ~ 0.814	0.133
College	0.433	0.132	0.602	0.140 ~ 1.592	0.095
Occupation					
Business and services	0.411	0.435	0.334	0.280 ~ 1.982	0.145
Farming	1.048	0.102	0.235	0.034 ~ 1.273	0.138
Others	0.392	0.137	0.549	0.247 ~ 1.952	0.199
Monthly income (RMB)					
< 3000	0.450	0.352	1.455	1.042 ~ 2.984	0.005
3000 ~ 6000	0.247	0.323	1.302	1.074 ~ 2.663	0.025
60,001 ~ 9000	0.341	0.254	0.826	0.459 ~ 1.754	0.092
> 9000	0.839	0.467	1.143	0.145 ~ 3.728	0.127

## Discussion

GDM is a disorder of glucose metabolism that occurs during pregnancy, and patients usually do not have any conscious manifestations. However, if the elevated blood sugar is not effectively controlled in time, the incidence of maternal and infant complications increase greatly [18]. Therefore, when most pregnant women with GDM are first diagnosed, pregnant women are required to become familiar with and master the measures for blood glucose monitoring and disease treatment as soon as possible. It has been reported that most GDM patients cannot cope with it in a short time [19]. Anxiety is a psychological disorder whose main characteristics are widespread and persistent anxiety and recurring panic [20]. Studies [21–23] have investigated the anxiety state of pregnant women with gestational diabetes, and the results show that up to 60.8% of women with GDM may have mild or more anxiety. The incidence of anxiety in patients with GDM in this present study is 59.07%, and we have found that for GDM patients with abnormal pregnancy and cesarean section history, first pregnancy, lower education level, and less monthly income, they have higher risk for anxiety, more attention and targeted interventions are needed for those patients.

Previous study [24] has shown that the incidence of stress and depression in GDM patients is 3.79 times that of normal pregnant women. In addition, studies [25, 26] have found that 66.3% of pregnant women’s anxiety comes from lack of understanding of their own diseases and related knowledge. The main anxiety problem is worry about fetal health [27]. The high glucose status of GDM patients will cause a series of adverse effects on the fetus [28]. Therefore, compared with normal pregnant women, GDM patients pay more attention to the health of the fetus, which causes great psychological pressure [29]. Furthermore, pregnancy-related anxiety level of women with first pregnancy is higher than that of multiparas, the potential reasons may be the fact that the primiparas have not experienced pregnancy or childbirth, and the uncertainty of illness effects on the health of the fetus.

The results of this study have found that social support, self-efficacy and pregnancy-related anxiety are negatively correlated, and the anxiety was positively correlated with susceptible personality. Social support refers to the supports that an individual obtains through social contact that can reduce psychological stress and improve social adaptability [30]. Pregnancy is a sensitive stage for women. Besides, the patient's psychology is more vulnerable due to GDM. At this stage, family members and friends should pay more attention to and help patients to relieve their anxiety. Self-efficacy is the content of positive psychology, which refers to the ability, judgment and beliefs to engage in a certain behavior and achieve expected results in a specific situation [31]. The stronger the self-efficacy of patients with GDM, the stronger their confidence in overcoming the disease, and the more proactively adopting disease response measures [32]. Therefore, it is necessary to help pregnant women improve their self-management ability and promote their own healthy behaviors.

The lower the education level and the lower the economic level of GDM patients, the higher their pregnancy-related anxiety level. The reasons for this finding may be that patients with higher education level and income have a wide range of platforms for obtaining knowledge about pregnancy and diabetes, and they are better at using surrounding social resources to obtain social support, which is beneficial to reduce patient negativity mood [33]. Besides, patients with a high level of education can better understand pregnancy and diabetes-related knowledge, and have better self-management capabilities, which are conducive to restraining their own unhealthy behaviors in daily life and adhere to the principles of GDM diet and exercise [34]. Higher household income is conducive to better coping with the economic expenses related to various inspections and medications in the course of disease treatment [35, 36].

GDM patients with a history of abnormal pregnancy and cesarean section history are prone to pregnancy-related anxiety [37]. For a patient with a history of abnormal pregnancy, they may involuntarily worry that a similar situation can happen again, thus becoming more sensitive, and often become overly nervous [38]. GDM patients with a history of abnormal pregnancy are more worried about fetal health than normal pregnant women [39]. Moreover, GDM can lead to a series of adverse pregnancy outcomes such as spontaneous abortion, abruption of the placenta, polyhydramnios, infection, premature delivery, etc. [40], which may make them oversensitive. Sensitive and nervous psychology causes more anxiety in patients and affects blood sugar control, and the poor blood sugar control aggravates anxiety in patients, which leads to a vicious cycle. Therefore, for patients with a history of abnormal pregnancy, special attention should be paid to relieve anxiety.

## Conclusions

In conclusion, the level of anxiety is relatively high in patients with GDM. Early nursing care and interventions are warranted for those patients with abnormal pregnancy and cesarean section history, first pregnancy, lower education level, and less monthly income to reduce the anxiety level. Early alert that they may have higher anxiety level in patients with those risk factors are needed in clinical practice. Medical staff should conduct reasonable pregnancy examinations and knowledge-rich health education for GDM patients. Besides, the family members, friends and colleagues should pay more attention to the anxiety emotions of GDM patients, patients should be encouraged to communicate with others more, and health care providers should help patients establish the health believes and behaviors to reduce the anxiety level of patients with GDM.

## Data Availability

We would like to share the raw data to the scholars who are interested in our study if requested, please contact Palida Maimaiti (email: parida0331@yeah.net) in that case.

## Abbreviations

GDM: Gestational diabetes mellitus
VPSQ: Vulnerable personality style questionnaire
GSES: General self-efficacy scale
SSRS: Social support rating scale
